# Iodine and Selenium Status in Relation to Thyroid and Immune Functions—The Analysis of Their Dependencies in a Group of Women of Reproductive Age from the Southern Region of Poland

**DOI:** 10.3390/nu17121952

**Published:** 2025-06-07

**Authors:** Jadwiga Kryczyk-Kozioł, Ewelina Prochownik, Justyna Dobrowolska-Iwanek, Paweł Paśko, Krzysztof Kleszcz, Renata Francik, Halina Potok, Magdalena Nieckula, Urszula Cisoń-Apanasewicz, Paulina Jabłońska, Dorota Ogonowska, Grażyna Kuzera, Mirosław Krośniak, Sanja Klobučar, Paweł Zagrodzki

**Affiliations:** 1 Department of Food Chemistry and Nutrition, Faculty of Pharmacy, Jagiellonian University Medical College, Medyczna 9, 30-688 Kraków, Poland; ewelina.gajdzik@uj.edu.pl (E.P.); justyna.dobrowolska-iwanek@uj.edu.pl (J.D.-I.); p.pasko@uj.edu.pl (P.P.); miroslaw.krosniak@uj.edu.pl (M.K.); 2 Faculty of Mathematics and Natural Sciences, Department of Chemistry, University of Applied Sciences in Tarnów, Mickiewicza 8, 33-100 Tarnow, Poland; k_kleszcz@atar.edu.pl; 3 Faculty of Medicine and Health Sciences, Academy of Applied Sciences in Nowy Sącz, Kościuszki 2G, 33-300 Nowy Sącz, Poland; rfrancik@ans-ns.edu.pl (R.F.); hpotok@ans-ns.edu.pl (H.P.); ucison-apanasewicz@ans-ns.edu.pl (U.C.-A.); dogonowska@ans-ns.edu.pl (D.O.); gkuzera@ans-ns.edu.pl (G.K.); 4 Medical Institute, Academy of Applied Sciences in Nowy Targ, Kokoszków 71, 34-400 Nowy Targ, Poland; magdalena.nieckula@ans-nt.edu.pl (M.N.); paulina.jablonska@ans-nt.edu.pl (P.J.); 5 Department of Endocrinology, Diabetes and Metabolic Diseases, Clinical Hospital Centre Rijeka, Faculty of Medicine, University of Rijeka, Krešimirova 42, 51000 Rijeka, Croatia; sanja.klobucar@uniri.hr

**Keywords:** iodine, selenium, thyroid gland, immune system, women, trace element, serum

## Abstract

**Objectives:** Iodine and selenium are key elements for thyroid. There is also evidence of their immunoregulatory potential. However, the current state of knowledge of potential interactions among iodine—selenium—thyroid—immune system is not sufficient. The aim of the study was to evaluate iodine and selenium statuses and examine the relationship between them and the functioning of the thyroid and immune system in a group of women of reproductive age, without previously diagnosed disease. **Methods:** The study involved a group of 60 women aged 19–40 from southern Poland. The concentrations of iodine and selenium were determined in serum samples using the ICP-MS and AAS methods, respectively. Thyroid function was assessed by determining serum levels of thyroid-stimulating hormone (TSH), free thyroxine (fT4), and anti-thyroid peroxidase antibodies (anti-TPO) by electrochemiluminescence methods. Glutathione peroxidase 3 (GPX3) and ferric ion reducing antioxidant power (FRAP) in serum were measured by spectrophotometric methods. Immune functions were evaluated by analyzing cytokine levels using ELISA tests, including interferon-γ, interleukin-4, interleukin-17, and transforming growth factor-β. **Results:** No significant correlations between selenium and thyroid or immunological parameters were observed. The level of iodine in serum positively correlated with free thyroxine, indicating its importance for maintaining normal thyroid function, as well as with FRAP in serum, suggesting a protective role of iodine-mediated antioxidant activity on thyroid function. **Conclusions:** Our results underline the complexity of the system of correlations between iodine–selenium–thyroid–immune function. Nevertheless, understanding them may turn out to be crucial for developing preventive and therapeutic strategies in the context of thyroid diseases.

## 1. Introduction

There is increasing evidence of interactions between the thyroid and immune system [1]. The role of iodine and selenium in thyroid function in euthyroid conditions has been well described in the literature [2,3]. However, their profile of biological properties is much broader than just an effect on this gland; one of them is concerned with modulating immune system functions [4,5].

Thyroid dysfunctions, which constitute frequent endocrine diseases, are more common among women. The results of our previous study prove that this problem also exists in Poland, where 17% of participants declared thyroid dysfunction. It is worth adding that 94% of attendees were people aged 18–40, and among them, 91.5% were women [6].

In Europe, the problem of iodine deficiency still occurs [7], and selenium intake is also considered inadequate [8]. Therefore, the main goal of our study was to evaluate iodine and selenium concentrations in serum, as well as an attempt to shed new light on the interplay among these trace elements and the thyroid and immune system functions in a group of women of reproductive age without previously diagnosed disease in a particular area of southern Poland, which historically belongs to endemic areas of goiter and may still be a region where cases of autoimmune thyroiditis are reported more often than in other parts of the country [9]. Importantly, in Poland (including the southern part), selenium supply was also insufficient [10,11]. Ioduria is a widely used marker of iodine resources in the human body; however, the collection of urine samples can be quite inconvenient for the patient. Therefore, in this study, we took the challenge to determine this element in serum, which was collected for the simultaneous determination of other parameters. To the best of the authors’ knowledge, this is a new approach in research in the Polish and European population.

## 2. Materials and Methods

### 2.1. Participants

The study involved women aged 19–40 years without previously diagnosed disease, living in the Małopolska region in the southern part of Poland. The recruitment process was conducted among inhabitants of Nowy Sącz and Nowy Targ and surrounding areas, between February and July 2021. The exclusion criteria were as follows: (1) permanent use of hormonal, immunomodulatory or iodine-containing drugs/preparations; (2) use of hormonal contraceptives (up to one year prior to recruitment); (3) previously diagnosed diseases; (4) smoking, alcohol abuse; (5) following special diets; (6) noticeable weight change; (7) pregnancy or lactation; (8) intensive sports practice.

### 2.2. Study Design

The study group consisted of 60 women. The blood samples from participants were taken in tubes containing a serum activator (VACUETTE^®^, Greiner Bio-One GmbH, Kremsmünster, Austria) (about 5–7 days before expected menstruation) and refrigerated for about 25 min, then centrifuged at +4 °C for 16 min at a speed of 3200 rpm. The obtained serum was transferred to tubes and stored at −80 °C for further analysis.

### 2.3. Chemicals

Ultrapure water (18.2 MΩ·cm) was obtained from Milli-Q Direct-Q^®^ 3 UV Water Purification-Merck-Millipore (Burlington, MA, USA). Iodine (100 mg/L) and selenium (1000 mg/L) standards were supplied by Sigma-Aldrich (St. Louis, Mo, USA) and Agilent (St. Clara, CA, USA), respectively. Certified reference material—SeronormTM Trace Elements Serum L-1 and ClinChek Serum Control (Level I) were purchased from SERO AS (Billingstad, Norway) and Recipe Chemicals (München, Germany), respectively. In addition, 25% tetramethylammonium hydroxide (TMAH), 69% nitric acid(V), palladium(II) nitrate (10 g Pd/L), magnesium nitrate (20 g/L), Triton X-100, sodium acetate, acetic acid, 2,4,6-tris(2-pyridyl)-s-triazine, 30% hydrochloric acid, iron(III) chloride hexahydrate, iron(II) sulfate heptahydrate, sodium phosphate monobasic dihydrate, sodium phosphate dibasic dihydrate, ethylenediaminetetraacetic acid disodium salt dihydrate, sodium azide, 30% hydrogen peroxide, glutathione reductase, reduced glutathione, β-nicotinamide adenine dinucleotide 2′-phosphate reduced tetrasodium salt hydrate were obtained from Sigma-Aldrich (St. Louis, MO, USA). Argon 4.8 and 5.0 were purchased from Air Liquide (Kraków, Poland). Kits for the assay of thyroid and immunological parameters (Human INF-γ ELISA KIT—E0105Hu, Human IL-4 ELISA KIT—EO0092Hu, Human IL-17A ELISA KIT—EO142Hu and Human TGF-β ELISA KIT—E3051Hu) were purchased from Roche Diagnostics GmbH, (Mannheim, Germany) and Bioassay Technology Laboratory (Zhejiang, China), respectively.

### 2.4. Determination of Iodine in Serum by ICP-MS

Serum samples were prepared as follows: 200 µL of tested sample and 200 µL TMAH were taken into a polypropylene tube (volume 15 mL) with screw cap. The tube was then secured with parafilm and left in a water bath (WB 22, MEMMERT, Schwabach, Germany) at +95 °C for 3 h. After cooling, 9.6 mL of deionized water was added, and then the solution was filtered using a nylon (PA) syringe filter, pore size 0.45 μm (Polygen, Gliwice, Poland). The prepared and secured samples were stored in the dark at +4 °C for 12 h until analysis. The analysis was performed on ICP-MS—SCIEX, ELAN^®^ DRC-e (PerkinElmer, Woodbridge, ON, Canada) and system operating parameters were the following: nebulizer gas flow—0.90 L/min; RF power—1100 W; plasma gas flow—15.0 L/min; cooling gas flow rate—17 L/min; reaction gas—argon. The analysis of biological samples using the ICP-MS method from patients was preceded by the determination of method validation parameters. Accuracy was estimated based on analysis of certified reference material—SeronormTM, which was reconstituted according to the manufacturer’s recommendations. The iodine concentration in the reference material was found to be 83.74 µg/L, and the manufacturer’s declared concentration value was 84 µg/L. The precision of determination expressed as relative standard deviation (RSD) was set at 7%. The developed method was also characterized by low limits of detection and quantification, 0.83 µg/L and 2.49 µg/L, respectively, and linearity of readings (R^2^ = 0.9967) over the range of concentrations of iodine standards (LOQ–200 µg/L) that were selected to prepare the calibration curve.

### 2.5. Determination of Selenium in Serum by AAS

As a matrix modifier, solution containing 5% HNO_3_, 1500 mg/L Pd, 6000 mg/L Mg(NO_3_)_2_ and 0.1% Triton X-100 was used. To ensure homogeneity, prior to analysis, each sample was shaken for 5 min using a vortex mixer. Additionally, samples were diluted with 0.1% Triton X-100 solution (150 µL of sample + 100 µL of Triton solution) to avoid possible phase separation or precipitation. The determination of selenium was performed using an Agilent 240Z AA atomic absorption spectrometer with graphite-furnace atomization and Zeeman background correction. A high-intensity hollow-cathode lamp (Agilent, St. Clara, CA, USA) was used as a light source. The instrument parameters were the following: wavelength: 196.0 nm, current: 12 mA, slit: 1.0 nm. The ashing and atomization temperatures were 950 °C and 2600 °C, respectively. In order to facilitate decomposition of the organic matrix, preliminary ashing with air as an auxiliary gas at 400 °C was included in the time/temperature program. The accuracy of the method was confirmed using certified reference material—ClinChek, which was reconstituted according to the manufacturer’s recommendations. Water was used as a blank sample (included in each series of samples), and based on its results, the limit of quantification was calculated. Analysis of serum reference material indicated that the analytical method developed was accurate, with the determined selenium concentration (59.9 µg/L) falling within the concentration range specified by the manufacturer (control range: 43.9–65.8 µg/L, certified value: 54.8 µg/L). In addition, the method was characterized by linearity of readings over the analyte concentration range used, with an R^2^ of 0.9950, high precision with an RSD of 4%, and relatively low limits of detection (7.1 µg/L) and quantification (14.3 µg/L). The linear range of the method for the determination of selenium in serum was LOQ–102 ug/L.

### 2.6. Determination of Glutathione Peroxidase 3 in Serum

The assays of GPX3 in serum samples were performed based on the modified method of Paglia and Valentine, according to the procedure described earlier [12].

### 2.7. Determination of Thyroid Parameters in Serum

Serum TSH, fT4, and anti-TPO were determined commercially at the Department of Laboratory Diagnostics, Rydygier’s Specialist Hospital in Kraków, Poland, through electrochemiluminescence methods using the analyzer Cobas 8000 (Roche Diagnostics GmbH, Mannheim, Germany). The reference values were 0.27–4.20 (mIU/mL), 12–22 (pmol/L), and <34 (IU/mL) for TSH, fT4, and anti-TPO, respectively.

### 2.8. Determination of Immunological Parameters in Serum

Immunological parameters such as interferon γ (INF-γ), interleukin 4 (IL-4), interleukin 17 (IL-17), and transforming growth factor β (TGF-β) in serum samples were determined using enzyme immunoassays according to the protocol provided by the manufacturer. The ranges of standard curves given by the producer were as follows: 1–400 pg/mL for INF-γ, 5–1000 pg/mL (after conversion from ng/L) for IL-4, 2–600 pg/mL (after conversion from ng/L for IL-17, 0.005–2 ng/mL (after conversion from pg/mL) for TGF-β. A modular multi-sensing microplate reader, Synergy 2 (BioTek Instruments, Winooski, VT, USA), was used to read absorbance values with the following parameters: wavelength: 450 nm, temperature: 22 °C.

### 2.9. Determination of FRAP in Serum

The method of Benzie and Strain was used to determine FRAP in serum, as described in our previous paper [13].

### 2.10. Statistical Approach

Descriptive statistics were calculated for all parameters in the whole study group. To calculate the mean values for parameters with non-Gaussian distribution, data were logarithmically transformed and retransformed after calculations. Having determined values of many biochemical parameters, it was decided to use the Principal Component Analysis (PCA) to reduce the number of parameters in an original data set while preserving as much information as possible [14]. The PCA model was used to show the correlation structure between parameters in the whole group of volunteers. The parameters with large absolute values of their coordinates (>0.3) on the first two principal components in the PCA model were assumed to determine the axes of the new coordinate system in PCA to the greatest extent. To express the strength of bivariate associations between such parameters, cosines of corresponding angles (i.e., correlation coefficients) were calculated. The “corresponding angle” means the angle determined by two lines connecting the origin with the coordinates of both parameters on the PCA loadings plot. The same approach was also used to check whether any clusters of volunteers or distant subjects appear in the PCA score plot and, if so, what is the reason for this. Statistical analyses were conducted using the STATISTICA v. 13.3. package (TIBCO Software Inc., Palo Alto, CA, USA).

## 3. Results

The mean ± SD of age of participants in the study was 25.1 ± 6.1 years, and BMI was 23.0 ± 3.7 kg/m^2^. The results of the determination of trace elements, i.e., iodine and selenium, GPX3, and FRAP, are summarized in Table 1, while markers of thyroid function, i.e., TSH, fT4, and anti-TPO, are shown in Table 2. The immunological parameters (i.e., IFN-γ, IL-4, IL-17, and TGF-β) are presented in Table 3. The outcomes are expressed as mean ± standard deviation or mean (confidence interval obtained after converting data to logarithms); median and range of values (minimum-maximum).

**Figure 1:**
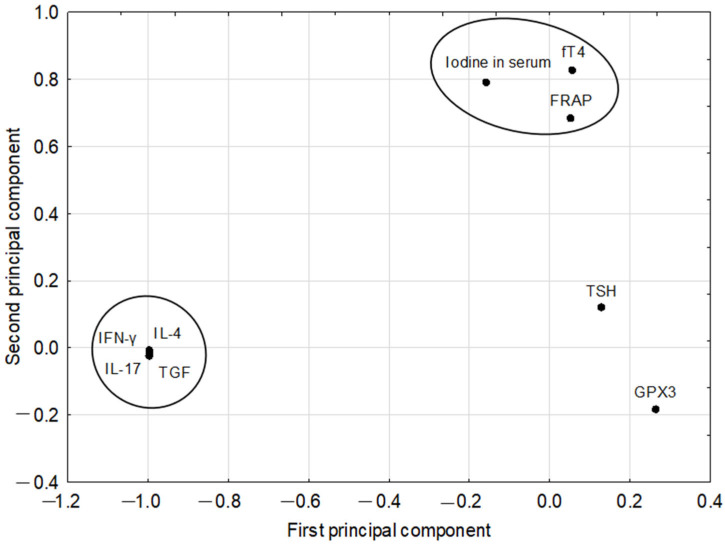
The original parameter loadings for the first two principal components (the clusters of strongly related parameters are enclosed by ellipses).

The PCA model was constructed for the parameters listed in the Statistical Approach section, and it had two significant components, with eigenvalues of 4.08 and 1.83, and explained 65.7% of the variance of the original parameters. The first principal component in this model had strong negative weights predominantly for IFN-γ, IL-4, IL-17, and TGF-β. All these parameters, being in one very tight cluster, were strongly correlated with each other. Whereas positive weights on the first component were below the absolute value of 0.3. The second principal component was loaded mainly positively by fT4, iodine in serum, and FRAP, which also showed strong mutual correlations, while parameters loading negatively on this component had low (GPX3) or very low (IFN-γ, IL-4, IL-17, TGF-β) absolute values of their weights. Other parameters (Se, anti-TPO) were not included in the model as they were considered noninformative, which means that they had very little contribution to the formation of principal components. The loadings for the first two principal components are shown in Figure 1. Correlation coefficients for pairs of parameters based on the PCA model were gathered in Table 4.

## 4. Discussion

Considering that a daily urine sample or 10-spot samples are needed to assess an individual’s content of this element [15], or that collecting a urine sample can be inconvenient, alternative biological materials, including blood [16], are becoming increasingly preferred. Thus, in this study, it was decided to measure the iodine concentration in blood samples both as a new alternative and a less burdensome method for participants. In addition, the level of iodine in serum is a more stable indicator reflecting the status of this element in an organism than iodine in urine, and to some extent, it reflects the functional state of the thyroid. Jin et al. [16] established the range of serum iodine concentration as 48.6–122.0 μg/L in the Chinese population of euthyroid adults. In turn, Yu et al. [17], based on a nationwide multicenter study of the Chinese euthyroid adult population, proposed a reference range of 36.0–79.3 μg/L. The mean serum iodine concentration in our study group (Table 1) was within or close to the ranges reported by the authors of the above-mentioned research. To the best of our knowledge, these are the first studies to determine serum iodine in the Polish and European populations.

The mean concentrations of the tested markers of thyroid function and anti-TPO (Table 2) were within reference values. The PCA model revealed a correlation between iodine in serum and fT4 (Table 4), which has also been noted by other authors [16] and provides further evidence of the close relationship between iodine and metabolism in the human body and the synthesis of thyroid hormones.

The effects of selenium on organisms are mediated by selenoproteins. GPX3 is one of them and takes part in the protection of cells against oxidative damage. Therefore, in addition to selenium, this protein was also determined (Table 1). Se concentrations in our study group were within the range of optimal values, i.e., 90–120 μg/L. Nevertheless, it is suggested that GPX3 peaks in activity at selenium levels of about 90 μg/L [18]. Interestingly, results of other studies conducted also among healthy Polish women in other areas are quite varied (i.e., in central Poland—Łódź: Se 57.0 ± 11.8 µg/L and GPX3 191 ± 32 U/L (after conversion from pg/mL) [19] vs. north-western Poland—Szczecin: Se 99.4 ± 19.7 µg/L [20]) and can be evidence of variations in its supply within geographically similar regions, even in the same country. In addition, age should also be mentioned as a factor that may be of relevance to the outcome. In the studies cited above, the mean age of participants was higher than in our present study. In a retrospective observational cohort study conducted in the general population in western Romania in 2019, it was observed that the highest percentage of participants with selenium level in serum in the reference range (i.e., 70–130 μg/L) was in 16–35 years group (i.e., 70.1%) and the lowest in 66–89 years group (i.e., 42.6%). The percentage of participants aged 36–65 having selenium concentrations within the aforementioned range was 64.7% [21]. In addition, the phase of the menstrual cycle can also affect the obtained values of markers of selenium status in an organism [22]. Taking this into account, in our study, blood samples were taken approximately 5–7 days before expected menstruation, whereas in cited research, this aspect was often omitted in the description of the study group.

The value of FRAP (Table 1) was similar to the results of other studies on healthy Polish groups [13]. FRAP did not show any correlation with GPX3, although both parameters in our study reflect the antioxidative potential of blood. Apparently, measurement of FRAP apart from GPX3 considers many other (also non-protein) components of blood serum that react differently than GPX3; therefore, there is no correlation between these parameters. However, because of this, both parameters are not mutually redundant and provide complementary information regarding antioxidant defense.

In our study, statistical analysis did not reveal any correlation between selenium parameters and thyroid parameters, similar to the results of Fontenelle et al. [23]. The impact of selenium on the metabolism of thyroid hormones is indirect, both through protection against oxidative damage (by glutathione peroxidases and thioredoxin reductases) and participation in activation/inactivation of these hormones by deiodinases. Therefore, the relationship between selenium and thyroid parameters, especially in relatively homeostatic conditions (i.e., young women without previously diagnosed disease), is not directly proportional. The positive correlation between FRAP and fT4 (Table 4) is in agreement with the results of Reddy et al. [24].

Autoimmune thyroid diseases (AITD), including HT, are some of the most common autoimmune disorders. This particular thyroid disease can affect up to 8.0% of the adult population in Europe, but women are about four times more prone [25]. Therefore, in our study, when selecting immunological parameters, lymphocyte disturbances usually observed in patients with HT were taken into account, i.e., Th1, Th2, and Th17 represented by INF-γ, IL-4, and IL-17, respectively, as well as Treg via TGF-β. The results for these parameters are summarized in Table 3. IFN-γ is a pro-inflammatory cytokine that stimulates a Th1-dependent immune response [26]. In current research, the obtained mean value of INF-γ (Table 3) was much higher than the results obtained in groups of healthy subjects (including both genders) by Matowicka-Karna et al. [27] in Poland (i.e., 7.0 ± 3.9 pg/mL), and lower than what was reported by Shi et al. [28] in China (i.e., 106.7 ± 8.4 pg/mL), or Tomaszewska et al. [29] in Poland (i.e., 199.6 ± 41.3 pg/mL).

Another cytokine analyzed was IL-4, which is involved in processes such as differentiation of naive T cells into Th2 cells, production of B lymphocyte antibodies, or differentiation of macrophages into anti-inflammatory type M2 [30]. The level of IL-4 among participants of the present study (Table 3) was higher than outcomes of other studies in healthy populations (including both genders): 1.77 pg/mL in Greece [31]; 0.55 ± 0.24 pg/mL in Poland [32]. In turn, IL-17 has been linked with chronic inflammation observed among others in the pathogenesis of AITD. The mean concentration of IL-17 in this study (Table 3) was higher compared to the result of Zajkowska et al. in Poland [32], 3.47 ± 2.65 pg/mL. On the other hand, our result was close to the one obtained by Shi et al. in China [28], 98.4 ± 5.5 pg/mL.

TGF-β is a cytokine that exerts anti-inflammatory and immunosuppressive effects [33]. The result obtained in the present study (Table 3) was much lower compared to Aleksandrova et al. in Bulgaria [34], 8.06 ± 4.07 ng/mL, but still higher than the results of Davami et al. in Iran [35], 0.17 ± 0.03 ng/mL (after conversion from pg/mL).

The variation in results among immunological parameters of the above-mentioned authors and ours is puzzling and difficult to interpret. Nevertheless, it is worth pointing out differences in methodology, such as blood fraction (serum or plasma), origin of patients (European or Asian population), and perhaps most likely reason, specificity of the test used for assessment.

The dispersion of our results may also provide evidence of the influence of interpersonal factors even among healthy people. In the light of results of Manolova et al. [36], it may be assumed that gender is an important factor in cytokine production because in the group of healthy women, the level of TGF-β was equal to 26.1 ± 17.3 ng/mL, while among men, it was much higher at 61.0 ± 31.3 ng/mL. But available studies often provide the mean value for the entire group without division by gender [27,28,29,31,32,34,35]. Age should also be taken into account (e.g., mean value was much higher and range of age was wider in the case of the group in the quoted paper, 62.1 ± 11.8; 25–82 years, respectively [34], which is unlike that in our group, i.e., 25.1 ± 6.1; 19–40 years), particularly because Manolova et al. [36] observed a statistical difference in the level of TGF-β between groups aged <45 and >45 years, i.e., 21.3 vs. 39.2 ng/mL. It is also worth highlighting that in our study, participants were inhabitants of a specific region of Poland (i.e., Małopolska), which increased homogeneity of this group in terms of ethnicity or environmental factors.

The PCA model applied to our data revealed significant correlations among the analyzed cytokines (Table 4). A positive correlation between INF-γ and IL-4 appears to be important in the context of auto-prevention of autoimmune thyroid diseases, e.g., in the course of HT, Th1 cell activity stimulated by INF-γ increases, which can be repressed by IL-4. On the other hand, overexpression of IL-4 in thyrocytes impairs iodide uptake [37] and increased the susceptibility to develop severe, accelerated, and spontaneous autoimmune thyroiditis in response to excessive iodide supply in an animal model of HT [38]. The results from observational studies provide evidence of the association between iodine intake and thyroid autoimmunity, indicating U-shaped dependence [39]. Cuenca-Micó et al. [40] in an ex vivo experiment observed an increase in expression of Th1 and Th17 pathways in the presence of iodine. In our study, these T-cell subpopulations were represented by INF-γ and IL-17, respectively, for which the PCA model also revealed a positive correlation (Table 3). However, the mean level of iodine in serum (Table 1) was close to or within the ranges reported by other authors [16,17], which may provide a partial explanation for the correlation between all analyzed cytokines, showing both pro- and anti-inflammatory effects but without such a dependence on iodine in serum (Table 4).

In meta-analysis [41], no evident impact of selenium supplementation on changes in the level of cytokine (e.g., IL-10, IFN-γ) was observed in a group of healthy people. Additionally, an increase in concentrations of Se in plasma above 100 μg/L had no influence on further elevation in IgA or T cell levels. The mean selenium concentration in the study group was 95.4 µg/L, so perhaps this is the reason why there was no correlation between this trace element (as well as GPX3) and the immunological parameters. Furthermore, there is still insufficient conclusive evidence on the association between selenium intake and immunity in the context of the general population.

## 5. Limitations of This Study

Although extensive efforts were made to constitute a relatively homogeneous group in order to reduce factors that could potentially disrupt the results (e.g., residents of one region of Poland, i.e., Małopolska, to minimize the impact of environmental and ethnic diversity; a narrow age range of participants, i.e., 18–40 years; one gender—women; one phase of the menstrual cycle, i.e., about 5–7 days before expected menstruation; similar health status, i.e., no regular medication; use of a similar diet, i.e., following special diets was one of the criteria for exclusion from the study), there are still limitations, i.e., relatively small sample size of participants, cross-sectional design, and lack of subclinical disease assessment. Taking these methodological limitations into account in future studies may allow for a more accurate understanding of the interplay among the thyroid–iodine–selenium–immune system.

Nevertheless, it should be emphasized that the relatively small sample size (n = 60) in our study did not weaken the results obtained by the PCA method (revealed correlations between parameters) because the correlation coefficients obtained refer to parameters that were previously normalized and largely free from noise. In this case, post-hoc power analysis (α = 0.05, total sample size = 60) showed a statistical power of 100%. However, the PCA model explained less than 70% of the variance in the original parameters. For the Pearson coefficients calculated for the original parameters, the statistical power was much lower in some cases and approached 61%, indicating an increased risk of making a Type II error.

## 6. Conclusions

This study highlights the complexity of the interactions among iodine/selenium, the thyroid, and the immune system. At the same time, it emphasizes the importance of limiting factors that could potentially affect the results of biochemical parameters, as individual variability in trace element status and immune responses in the healthy group may be wider than previously thought. The lack of direct correlation between selenium and the thyroid parameter under homeostatic conditions suggests a more complex modulation of thyroid function by selenium. In turn, the positive correlation between iodine and the thyroid parameter underscores once again the critical importance of adequate iodine intake. The lack of association between Se, iodine in serum, and immunological parameters could also be due to the young age of the women, the relatively low number of patients, and the single point of the study. However, if it is true that autoimmunity is triggered by high iodine levels (and such cases also occurred in our study), then these results are somewhat surprising. Thus, this research provides additional insights into the roles of selenium and iodine in modulating both thyroid and immune functions in a population of women without previously diagnosed disease of reproductive age in Poland living in a region previously exposed to iodine deficiency that may currently have a higher incidence of autoimmune thyroiditis than in other parts of this country.

## Figures and Tables

**Table 1:** Iodine, selenium status parameters, and antioxidant activity in the serum of participants.

Parameter	Mean ± SD	Median (Min.–Max.)
Iodine (μg/L)	92.3 ± 14.6	91.0 (69.5–159.6)
Selenium (μg/L)	95.4 ± 17.2	91.2 (65.3–148.1)
GPX3 (U/L)	161.2 ± 23.4	160.7 (107.4–210.2)
FRAP (μmolFe^2+^/L)	750.5 ± 89.6	746.7 (545.7–979.1)

**Table 2:** Thyroid hormones and anti-thyroid antibodies in the serum of participants.

Parameter	Mean ± SD ^1^/Mean (CI) ^2^	Median (Min.–Max.)
TSH (mIU/mL)	2.5 ± 1.1	2.4 (0.8–6.0)
fT4 (pmol/L)	16.7 ± 2.6	16.7 (10.3–20.6)
anti-TPO (IU/mL)	9.2 (4.4–19.2)	6.4 (6.4–307.0)

^1^ Regarding parameters: TSH and fT4. ^2^ Regarding parameter: anti-TPO; mean and confidence interval obtained after converting data to logarithms.

**Table 3:** Immunological parameters in the serum of participants.

Parameter	Mean (CI) ^1^	Median (Min.–Max.)
INF-γ (pg/mL)	72.8 (35.3–149.9)	53.6 (20.6–300.7)
IL-4 (pg/mL)	183.9 (84.9–398.3)	135.3 (54.7–867.2)
IL-17 (pg/mL)	105.7 (53.9–207.0)	77.0 (44.0–399.8)
TGF-β (ng/mL)	0.67 (0.30–1.50)	0.47 (0.21–3.37)

^1^ Mean and confidence interval obtained after converting data to logarithms.

**Table 4:** Correlation coefficients for pairs of parameters based on the PCA model and Pearson correlation coefficients for pairs of original parameters.

Pairs of Correlated Parameters		Correlation Coefficients Based on PCA Model	Correlation Coefficients of Raw Parameters
fT4	FRAP	1.0000	0.367; *p* = 0.004
IL-17	TGF-β	1.0000	0.995; *p* = 0.000
IL-4	INF-γ	1.0000	0.996; *p* = 0.000
TGF-β	INF-γ	1.0000	0.995; *p* = 0.000
IL-17	INF-γ	1.0000	0.997; *p* = 0.000
IL-4	TGF-β	1.0000	0.997; *p* = 0.000
IL-4	IL-17	1.0000	0.996; *p* = 0.000
fT4	Iodine in serum	0.9649	0.544; *p* = 0.000
FRAP	Iodine in serum	0.9632	0.284; *p* = 0.028

## Data Availability

The raw data supporting the conclusions presented in this article will be made available by the authors upon request. The dataset is currently part of ongoing analyses and future publications. To preserve the integrity and novelty of the research, we are temporarily restricting public access to the full dataset.
